# (*E*)-*N*-(3,3-Diphenyl­allyl­idene)-3-nitro­aniline

**DOI:** 10.1107/S1600536812040354

**Published:** 2012-09-29

**Authors:** Joo Hwan Cha, Yong Koo Kang, Yong Seo Cho, Jae Kyun Lee, Jae Choon Woo

**Affiliations:** a Advanced Analysis Center, Korea Institute of Science & Technology, Hwarangro 14-gil, Seongbuk-gu, Seoul 136-791, Republic of Korea; b Center for Neuro-Medicine, Korea Institute of Science & Technology, Hwarangro 14-gil, Seongbuk-gu, Seoul 136-791, Republic of Korea; c Drug Discovery Platform Technology Team, Korea Research Institute of Chemical Technology, PO Box 107, Yuseong, Daejeon 305-600, Republic of Korea

## Abstract

In the title compound, C_21_H_16_N_2_O_2_, the 3-nitro­phenyl and two phenyl rings are twisted from the mean plane of the enimino fragment by 44.4 (1), 37.2 (1) and 74.1 (1)°, respectively. The crystal packing exhibits no classical inter­molecular contacts.

## Related literature

For the structure of (*E*)-*N*-(3,3-diphenylallylidene)-4-nitroaniline, see: Kang *et al.* (2012 ▶). For the crystal structures of other closely related compounds, see: Khalaji *et al.* (2008*a* ▶,*b* ▶).
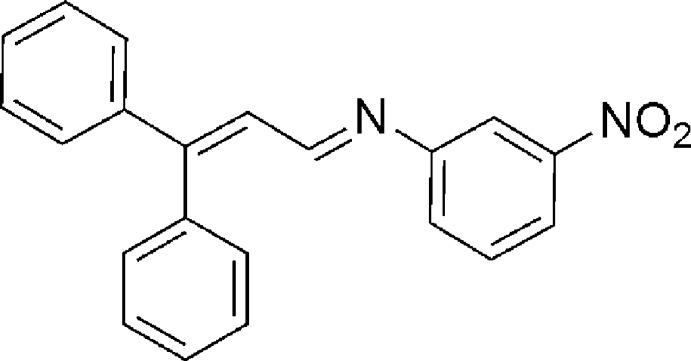


## Experimental

### 

#### Crystal data

C_21_H_16_N_2_O_2_
*M* *_r_* = 328.36
Monoclinic, 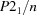

*a* = 5.8625 (7) Å
*b* = 22.825 (3) Å
*c* = 12.6370 (17) Å
β = 94.772 (4)°
*V* = 1685.1 (4) Å^3^
*Z* = 4
Mo *K*α radiation
μ = 0.08 mm^−1^
*T* = 296 K
0.40 × 0.20 × 0.20 mm

#### Data collection

Rigaku R-AXIS RAPID diffractometer
Absorption correction: multi-scan (*ABSCOR*; Rigaku, 1995 ▶) *T*_min_ = 0.967, *T*_max_ = 0.983
16270 measured reflections
3866 independent reflections
2663 reflections with *I* > 2σ(*I*)
*R*_int_ = 0.030

#### Refinement

*R*[*F* ^2^ > 2σ(*F* ^2^)] = 0.043
*wR*(*F* ^2^) = 0.136
*S* = 1.12
3866 reflections
226 parameters
H-atom parameters constrained
Δρ_max_ = 0.18 e Å^−3^
Δρ_min_ = −0.25 e Å^−3^

### 

Data collection: *RAPID-AUTO* (Rigaku, 2006 ▶); cell refinement: *RAPID-AUTO*; data reduction: *RAPID-AUTO*; program(s) used to solve structure: *SHELXS97* (Sheldrick, 2008 ▶); program(s) used to refine structure: *SHELXL97* (Sheldrick, 2008 ▶); molecular graphics: *CrystalStructure* (Rigaku, 2010 ▶); software used to prepare material for publication: *CrystalStructure*.

## Supplementary Material

Crystal structure: contains datablock(s) I, global. DOI: 10.1107/S1600536812040354/cv5343sup1.cif

Structure factors: contains datablock(s) I. DOI: 10.1107/S1600536812040354/cv5343Isup2.hkl

Supplementary material file. DOI: 10.1107/S1600536812040354/cv5343Isup3.cml

Additional supplementary materials:  crystallographic information; 3D view; checkCIF report

## Figures and Tables

**Fig. 1.**
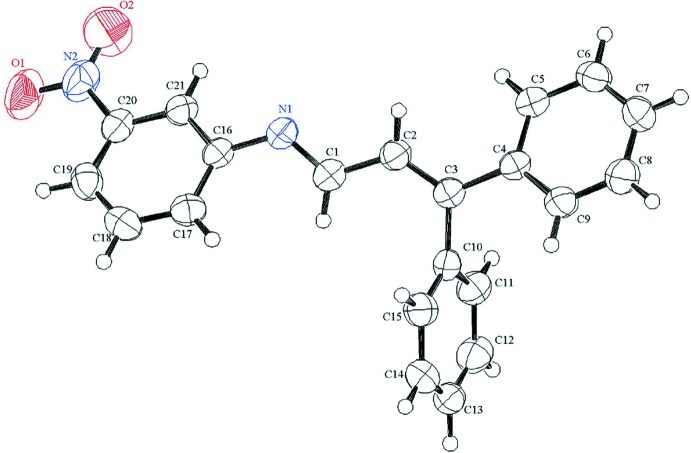
The molecular structure of the title compound showing the atomic numbering and 50% probability displacement ellipsoid.

## supplementary crystallographic information

### Comment

In the title molecule (Fig. 1), the dihedral angles between the mean planes of the central 3-nitrophenyl ring and phenyl rings C7–C12 and C1–C6 are 86.76 (8)° and 8.23 (3)°, respectively. The bond lengths and angles are unexceptional and correspond to those observed in the related compounds Khalaji *et al.* (2008**a*,b*). The imine group displays a torsion angle C21–C16–N1–C15 of -45.19 (18)°. The nitro group is twisted at 1.1 (2)° from the attached benzene ring. The crystal packing exhibits no classical intermolecular contacts.

### Experimental

To a solution of 3-nitroaniline (4.0 mmol) in ethanol (10 mL) was treated with equimolar quantities of substituted 2-phenylcinnamaldehydes. The mixture was refluxed for 5 h, and the progress of the reaction was monitored by TLC. Upon completion, the solvent was removed under reduced pressure. The residue was purified by flash column chromatography to afford the title compound in 60% yield. Recrystallization from ethanol gave crystals suitable for X-ray analysis.

### Refinement

All hydrogen atoms were positioned geometrically (C—H = 0.93 Å), and refined using a riding model, with *U*_iso_(H) = 1.2 *U*_eq_(C).

### Crystal data

**Table:**

C_21_H_16_N_2_O_2_	*F*(000) = 688
*M**_r_* = 328.36	*D*_x_ = 1.294 Mg m^−^^3^
Monoclinic, *P*2_1_/*n*	Mo *K*α radiation, λ = 0.71075 Å
Hall symbol: -P 2yn	Cell parameters from 8104 reflections
*a* = 5.8625 (7) Å	θ = 3.1–27.5°
*b* = 22.825 (3) Å	µ = 0.08 mm^−^^1^
*c* = 12.6370 (17) Å	*T* = 296 K
β = 94.772 (4)°	Block, colourless
*V* = 1685.1 (4) Å^3^	0.40 × 0.20 × 0.20 mm
*Z* = 4	

### Data collection

**Table:**

Rigaku R-AXIS RAPID diffractometer	3866 independent reflections
Radiation source: fine-focus sealed tube	2663 reflections with *I* > 2σ(*I*)
Graphite monochromator	*R*_int_ = 0.030
Detector resolution: 10.000 pixels mm^-1^	θ_max_ = 27.5°, θ_min_ = 3.1°
ω scans	*h* = −7→7
Absorption correction: multi-scan (*ABSCOR*; Rigaku, 1995)	*k* = −29→29
*T*_min_ = 0.967, *T*_max_ = 0.983	*l* = −16→16
16270 measured reflections	

### Refinement

**Table:**

Refinement on *F*^2^	Primary atom site location: structure-invariant direct methods
Least-squares matrix: full	Secondary atom site location: difference Fourier map
*R*[*F*^2^ > 2σ(*F*^2^)] = 0.043	Hydrogen site location: inferred from neighbouring sites
*wR*(*F*^2^) = 0.136	H-atom parameters constrained
*S* = 1.12	*w* = 1/[σ^2^(*F*_o_^2^) + (0.075*P*)^2^] where *P* = (*F*_o_^2^ + 2*F*_c_^2^)/3
3866 reflections	(Δ/σ)_max_ = 0.001
226 parameters	Δρ_max_ = 0.18 e Å^−^^3^
0 restraints	Δρ_min_ = −0.25 e Å^−^^3^

### Special details

**Table:**

Geometry. All esds (except the esd in the dihedral angle between two l.s. planes) are estimated using the full covariance matrix. The cell esds are taken into account individually in the estimation of esds in distances, angles and torsion angles; correlations between esds in cell parameters are only used when they are defined by crystal symmetry. An approximate (isotropic) treatment of cell esds is used for estimating esds involving l.s. planes.
Refinement. Refinement of F^2^ against ALL reflections. The weighted R-factor wR and goodness of fit S are based on F^2^, conventional R-factors R are based on F, with F set to zero for negative F^2^. The threshold expression of F^2^ > 2sigma(F^2^) is used only for calculating R-factors(gt) etc. and is not relevant to the choice of reflections for refinement. R-factors based on F^2^ are statistically about twice as large as those based on F, and R- factors based on ALL data will be even larger.

### Fractional atomic coordinates and isotropic or equivalent isotropic displacement parameters (Å^2^)

**Table:**

	*x*	*y*	*z*	*U*_iso_*/*U*_eq_	
O1	1.2806 (3)	0.79532 (9)	0.84554 (13)	0.1205 (6)	
O2	1.5869 (3)	0.74640 (6)	0.83527 (11)	0.0942 (5)	
N1	1.14274 (19)	0.90554 (5)	0.51096 (9)	0.0464 (3)	
N2	1.4414 (3)	0.78014 (6)	0.79813 (11)	0.0690 (4)	
C1	0.5605 (3)	1.01496 (6)	0.15119 (11)	0.0525 (4)	
H1	0.6562	1.0034	0.0999	0.063*	
C2	0.3920 (3)	1.05589 (7)	0.12595 (13)	0.0613 (4)	
H2	0.3776	1.0724	0.0584	0.074*	
C3	0.2448 (3)	1.07259 (7)	0.19989 (13)	0.0573 (4)	
H3	0.1292	1.0996	0.1820	0.069*	
C4	0.2700 (2)	1.04891 (6)	0.30091 (12)	0.0506 (3)	
H4	0.1717	1.0602	0.3513	0.061*	
C5	0.4406 (2)	1.00859 (6)	0.32705 (11)	0.0452 (3)	
H5	0.4567	0.9931	0.3953	0.054*	
C6	0.5893 (2)	0.99065 (5)	0.25294 (10)	0.0405 (3)	
C7	1.0213 (2)	0.90953 (7)	0.14288 (12)	0.0557 (4)	
H7	1.1302	0.9376	0.1645	0.067*	
C8	1.0584 (3)	0.87204 (8)	0.05913 (13)	0.0639 (4)	
H8	1.1913	0.8756	0.0243	0.077*	
C9	0.9005 (3)	0.82994 (7)	0.02771 (12)	0.0604 (4)	
H9	0.9265	0.8050	−0.0282	0.072*	
C10	0.7044 (3)	0.82455 (6)	0.07869 (13)	0.0594 (4)	
H10	0.5979	0.7957	0.0580	0.071*	
C11	0.6648 (2)	0.86225 (6)	0.16137 (11)	0.0510 (3)	
H11	0.5306	0.8587	0.1953	0.061*	
C12	0.8224 (2)	0.90501 (6)	0.19400 (10)	0.0410 (3)	
C13	0.7721 (2)	0.94683 (5)	0.28026 (10)	0.0398 (3)	
C14	0.8838 (2)	0.94559 (6)	0.37731 (10)	0.0442 (3)	
H14	0.8489	0.9749	0.4245	0.053*	
C15	1.0533 (2)	0.90295 (6)	0.41509 (10)	0.0443 (3)	
H15	1.0975	0.8737	0.3699	0.053*	
C16	1.3099 (2)	0.86430 (5)	0.54794 (10)	0.0410 (3)	
C17	1.2973 (2)	0.84251 (6)	0.65043 (10)	0.0463 (3)	
H17	1.1783	0.8536	0.6904	0.056*	
C18	1.4632 (2)	0.80448 (6)	0.69138 (11)	0.0482 (3)	
C19	1.6476 (2)	0.78813 (6)	0.63736 (13)	0.0539 (4)	
H19	1.7594	0.7630	0.6678	0.065*	
C20	1.6606 (2)	0.81029 (6)	0.53643 (13)	0.0554 (4)	
H20	1.7835	0.8002	0.4981	0.067*	
C21	1.4928 (2)	0.84741 (6)	0.49177 (11)	0.0495 (3)	
H21	1.5026	0.8613	0.4231	0.059*	

### Atomic displacement parameters (Å^2^)

**Table:**

	*U*^11^	*U*^22^	*U*^33^	*U*^12^	*U*^13^	*U*^23^
O1	0.1312 (13)	0.1592 (16)	0.0769 (10)	0.0438 (12)	0.0438 (10)	0.0502 (10)
O2	0.1215 (11)	0.0826 (9)	0.0741 (9)	0.0176 (8)	−0.0187 (8)	0.0249 (7)
N1	0.0500 (6)	0.0493 (6)	0.0393 (6)	0.0027 (5)	0.0002 (5)	0.0003 (5)
N2	0.0866 (10)	0.0658 (9)	0.0528 (8)	0.0040 (7)	−0.0050 (8)	0.0130 (7)
C1	0.0609 (8)	0.0564 (8)	0.0408 (7)	0.0120 (7)	0.0083 (6)	0.0034 (6)
C2	0.0769 (10)	0.0594 (9)	0.0473 (8)	0.0162 (8)	0.0031 (7)	0.0086 (7)
C3	0.0589 (8)	0.0508 (8)	0.0614 (9)	0.0128 (7)	0.0000 (7)	−0.0032 (7)
C4	0.0492 (7)	0.0508 (8)	0.0528 (8)	0.0019 (6)	0.0092 (6)	−0.0097 (6)
C5	0.0489 (7)	0.0467 (7)	0.0402 (7)	−0.0042 (6)	0.0051 (6)	−0.0033 (6)
C6	0.0439 (6)	0.0399 (6)	0.0375 (6)	−0.0028 (5)	0.0025 (5)	−0.0023 (5)
C7	0.0442 (7)	0.0690 (9)	0.0536 (8)	−0.0002 (7)	0.0027 (6)	−0.0100 (7)
C8	0.0522 (8)	0.0849 (11)	0.0549 (9)	0.0185 (8)	0.0053 (7)	−0.0081 (8)
C9	0.0762 (10)	0.0566 (9)	0.0464 (8)	0.0275 (8)	−0.0068 (8)	−0.0081 (7)
C10	0.0752 (10)	0.0443 (8)	0.0564 (9)	0.0008 (7)	−0.0073 (8)	−0.0059 (6)
C11	0.0552 (8)	0.0493 (8)	0.0483 (8)	−0.0040 (6)	0.0020 (6)	−0.0015 (6)
C12	0.0420 (6)	0.0421 (7)	0.0380 (7)	0.0047 (5)	−0.0019 (5)	0.0020 (5)
C13	0.0409 (6)	0.0403 (6)	0.0386 (6)	−0.0033 (5)	0.0051 (5)	0.0012 (5)
C14	0.0474 (7)	0.0443 (7)	0.0405 (7)	−0.0004 (6)	0.0010 (6)	−0.0019 (5)
C15	0.0479 (7)	0.0448 (7)	0.0400 (7)	−0.0032 (6)	0.0020 (6)	−0.0004 (5)
C16	0.0439 (6)	0.0415 (6)	0.0370 (6)	−0.0037 (5)	−0.0001 (5)	−0.0026 (5)
C17	0.0477 (7)	0.0519 (7)	0.0395 (7)	0.0006 (6)	0.0053 (6)	−0.0013 (6)
C18	0.0559 (7)	0.0451 (7)	0.0425 (7)	−0.0035 (6)	−0.0034 (6)	0.0011 (6)
C19	0.0528 (8)	0.0434 (7)	0.0637 (9)	0.0046 (6)	−0.0056 (7)	−0.0020 (6)
C20	0.0485 (7)	0.0523 (8)	0.0667 (10)	0.0017 (7)	0.0123 (7)	−0.0082 (7)
C21	0.0544 (7)	0.0502 (7)	0.0450 (7)	−0.0032 (6)	0.0094 (6)	−0.0004 (6)

### Geometric parameters (Å, º)

**Table:**

O1—N2	1.209 (2)	C9—C10	1.369 (2)
O2—N2	1.2144 (18)	C9—H9	0.9300
N1—C15	1.2808 (16)	C10—C11	1.388 (2)
N1—C16	1.4101 (16)	C10—H10	0.9300
N2—C18	1.4742 (19)	C11—C12	1.3831 (19)
C1—C2	1.378 (2)	C11—H11	0.9300
C1—C6	1.3977 (18)	C12—C13	1.4965 (18)
C1—H1	0.9300	C13—C14	1.3419 (18)
C2—C3	1.378 (2)	C14—C15	1.4435 (18)
C2—H2	0.9300	C14—H14	0.9300
C3—C4	1.383 (2)	C15—H15	0.9300
C3—H3	0.9300	C16—C21	1.3890 (19)
C4—C5	1.3792 (19)	C16—C17	1.3950 (18)
C4—H4	0.9300	C17—C18	1.3722 (19)
C5—C6	1.3930 (18)	C17—H17	0.9300
C5—H5	0.9300	C18—C19	1.377 (2)
C6—C13	1.4852 (17)	C19—C20	1.380 (2)
C7—C12	1.383 (2)	C19—H19	0.9300
C7—C8	1.392 (2)	C20—C21	1.383 (2)
C7—H7	0.9300	C20—H20	0.9300
C8—C9	1.370 (2)	C21—H21	0.9300
C8—H8	0.9300		
			
C15—N1—C16	120.07 (12)	C12—C11—C10	120.85 (14)
O1—N2—O2	122.82 (15)	C12—C11—H11	119.6
O1—N2—C18	118.43 (14)	C10—C11—H11	119.6
O2—N2—C18	118.75 (16)	C7—C12—C11	118.79 (12)
C2—C1—C6	120.77 (13)	C7—C12—C13	121.17 (12)
C2—C1—H1	119.6	C11—C12—C13	119.99 (11)
C6—C1—H1	119.6	C14—C13—C6	120.97 (12)
C3—C2—C1	120.55 (14)	C14—C13—C12	122.78 (11)
C3—C2—H2	119.7	C6—C13—C12	116.25 (10)
C1—C2—H2	119.7	C13—C14—C15	125.94 (12)
C2—C3—C4	119.55 (13)	C13—C14—H14	117.0
C2—C3—H3	120.2	C15—C14—H14	117.0
C4—C3—H3	120.2	N1—C15—C14	119.87 (13)
C5—C4—C3	120.11 (13)	N1—C15—H15	120.1
C5—C4—H4	119.9	C14—C15—H15	120.1
C3—C4—H4	119.9	C21—C16—C17	118.56 (12)
C4—C5—C6	121.14 (13)	C21—C16—N1	124.11 (12)
C4—C5—H5	119.4	C17—C16—N1	117.18 (12)
C6—C5—H5	119.4	C18—C17—C16	119.03 (13)
C5—C6—C1	117.87 (12)	C18—C17—H17	120.5
C5—C6—C13	121.42 (12)	C16—C17—H17	120.5
C1—C6—C13	120.71 (12)	C17—C18—C19	123.04 (13)
C12—C7—C8	120.03 (14)	C17—C18—N2	118.09 (14)
C12—C7—H7	120.0	C19—C18—N2	118.87 (13)
C8—C7—H7	120.0	C18—C19—C20	117.71 (13)
C9—C8—C7	120.46 (15)	C18—C19—H19	121.1
C9—C8—H8	119.8	C20—C19—H19	121.1
C7—C8—H8	119.8	C21—C20—C19	120.63 (14)
C10—C9—C8	120.01 (14)	C21—C20—H20	119.7
C10—C9—H9	120.0	C19—C20—H20	119.7
C8—C9—H9	120.0	C20—C21—C16	120.99 (13)
C9—C10—C11	119.85 (14)	C20—C21—H21	119.5
C9—C10—H10	120.1	C16—C21—H21	119.5
C11—C10—H10	120.1		
			
C6—C1—C2—C3	−1.6 (2)	C7—C12—C13—C6	107.60 (13)
C1—C2—C3—C4	1.4 (2)	C11—C12—C13—C6	−69.96 (15)
C2—C3—C4—C5	−0.4 (2)	C6—C13—C14—C15	175.77 (12)
C3—C4—C5—C6	−0.4 (2)	C12—C13—C14—C15	−4.3 (2)
C4—C5—C6—C1	0.18 (18)	C16—N1—C15—C14	−179.44 (11)
C4—C5—C6—C13	−179.42 (11)	C13—C14—C15—N1	−177.81 (13)
C2—C1—C6—C5	0.8 (2)	C15—N1—C16—C21	45.24 (18)
C2—C1—C6—C13	−179.58 (13)	C15—N1—C16—C17	−139.32 (13)
C12—C7—C8—C9	−1.0 (2)	C21—C16—C17—C18	−1.14 (18)
C7—C8—C9—C10	0.1 (2)	N1—C16—C17—C18	−176.83 (11)
C8—C9—C10—C11	0.7 (2)	C16—C17—C18—C19	2.3 (2)
C9—C10—C11—C12	−0.7 (2)	C16—C17—C18—N2	−177.55 (12)
C8—C7—C12—C11	1.0 (2)	O1—N2—C18—C17	−1.1 (2)
C8—C7—C12—C13	−176.60 (13)	O2—N2—C18—C17	179.95 (14)
C10—C11—C12—C7	−0.2 (2)	O1—N2—C18—C19	179.04 (17)
C10—C11—C12—C13	177.46 (12)	O2—N2—C18—C19	0.1 (2)
C5—C6—C13—C14	−36.10 (17)	C17—C18—C19—C20	−1.6 (2)
C1—C6—C13—C14	144.31 (14)	N2—C18—C19—C20	178.26 (12)
C5—C6—C13—C12	143.96 (12)	C18—C19—C20—C21	−0.3 (2)
C1—C6—C13—C12	−35.63 (16)	C19—C20—C21—C16	1.4 (2)
C7—C12—C13—C14	−72.33 (17)	C17—C16—C21—C20	−0.66 (19)
C11—C12—C13—C14	110.11 (14)	N1—C16—C21—C20	174.72 (11)
